# Pharmacogenomic Characterization and Isobologram Analysis of the Combination of Ascorbic Acid and Curcumin—Two Main Metabolites of *Curcuma longa*—in Cancer Cells

**DOI:** 10.3389/fphar.2017.00038

**Published:** 2017-02-02

**Authors:** Edna Ooko, Onat Kadioglu, Henry J. Greten, Thomas Efferth

**Affiliations:** ^1^Department of Pharmaceutical Biology, Institute of Pharmacy and Biochemistry, Johannes Gutenberg UniversityMainz, Germany; ^2^Heidelberg School of Chinese MedicineHeidelberg, Germany; ^3^Abel Salazar Biomedical Sciences Institute, University of PortoPorto, Portugal

**Keywords:** drug interaction, isobologram analysis, pharmacogenomics, phytotherapy, synergism

## Abstract

*Curcuma longa* has long been used in China and India as anti-inflammatory agent to treat a wide variety of conditions and also as a spice for varied curry preparations. The chemoprofile of the *Curcuma* species exhibits the presence of varied phytochemicals with curcumin being present in all three species but AA only being shown in *C. longa*. This study explored the effect of a curcumin/AA combination on human cancer cell lines. The curcumin/AA combination was assessed by isobologram analysis using the Loewe additivity drug interaction model. The drug combination showed additive cytotoxicity toward CCRF-CEM and CEM/ADR5000 leukemia cell lines and HCT116p53^+/+^ and HCT116p53^−/−^ colon cancer cell line, while the glioblastoma cell lines U87MG and U87MG.ΔEGFR showed additive to supra-additive cytotoxicity. Gene expression profiles predicting sensitivity and resistance of tumor cells to induction by curcumin and AA were determined by microarray-based mRNA expressions, COMPARE, and hierarchical cluster analyses. Numerous genes involved in transcription (*TFAM, TCERG1, RGS13, C11orf31*), apoptosis-regulation (*CRADD, CDK7, CDK19, CD81, TOM1*) signal transduction (*NR1D2, HMGN1, ABCA1, DE4ND4B, TRIM27*) DNA repair (*TOPBP1, RPA2*), mRNA metabolism (*RBBP4, HNRNPR, SRSF4, NR2F2, PDK1, TGM2*), and transporter genes (*ABCA1*) correlated with cellular responsiveness to curcumin and ascorbic acid. In conclusion, this study shows the effect of the curcumin/AA combination and identifies several candidate genes that may regulate the response of varied cancer cells to curcumin and AA.

## Introduction

*Curcuma longa* L. belongs to the family Zingiberaceae, which is a perennial herb that measures up to 1 m height with a short stem. It is distributed throughout tropical and subtropical regions in the world, being widely cultivated in Asian countries, mainly in India and China (Kapoor, 1990). As a component of folklore medicine the use of *C. longa* has been documented both in Indian and Chinese cultures. The rhizomes are used as *Ezhu* according to the Chinese Pharmacopoeia (2010 edition; Zhao et al., 2010) and are a household remedy in Nepal (Eigner and Scholz, 1999). The long list of usages include antiseptic, analgesic, anti-inflammatory, antimalarial, and insect repellant (Chaudhri, 1950; Li et al., 1998; Niederau and Göpfert, 1999; 2001; Tawatsin et al., 2001; Duke, 2002). Traditional Indian medicine claims to use its powder against biliary disorders, anorexia, coryza, cough, diabetic wounds, hepatic disorders, rheumatism, and sinusitis (Ammon et al., 1992). In ancient Hindu medicine, *C. longa* is extensively used for the treatment of sprains and swelling caused by injury (Ammon and Wahl, 1991). The antioxidant activity of *C. longa* is well-known (Ammon and Wahl, 1991; Anand et al., 2007).

Extensive research during the past years revealed that curcumin has considerable potential against a wide variety of both malignant and non-malignant diseases. Curcumin exhibits activity against numerous inflammatory diseases, including pancreatitis (Gukovsky et al., 2003; Gülçubuk et al., 2005), arthritis (Joe et al., 1997; Liacini et al., 2002), inflammatory bowel disease (Holt et al., 2005), gastritis (Swarnakar et al., 2005), allergy (Baek et al., 2003; Ram et al., 2003), and fever (Lee et al., 2003; Shao et al., 2004), possibly through the downregulation of inflammatory markers. It is also active against autoimmune diseases, including scleroderma (Tourkina et al., 2004), psoriasis (Bosman, 1994), multiple sclerosis (Verbeek et al., 2005), and diabetes (Babu and Srinivasan, 1995, 1997; Sajithlal et al., 1998). Curcumin also exhibits a great potential against various types of cancers. Its mechanism of action involves, firstly, the suppression of tumor cell proliferation by down-regulation of anti-apoptotic gene products, activation of caspases, and induction of tumor suppressor genes (Jiang et al., 1996; Bush et al., 2001; Chan and Wu, 2004). Secondly, curcumin suppresses tumor invasion by down-regulation of matrix metalloproteinases and cell surface adhesion molecules (Lin et al., 1998; Fenton et al., 2002; Lee et al., 2006). Thirdly, curcumin inhibits angiogenic cytokines leading to suppression of angiogenesis (Shin et al., 2001; Leyon and Kuttan, 2003; Bobrovnikova-Marjon et al., 2004) and lastly the anti-inflammatory and cytotoxic effects of curcumin contribute to its antitumor activity (Srivastava, 1989; Fujiyama-Fujiwara et al., 1992; Ammon et al., 1993).

The activity of many medicinal plants results from the interaction action of several constituents, which may cooperatively act in an additive or synergistic manner. It has been repeatedly observed that extracts of medicinal plants reveal better activities than their isolated single compounds at comparable equivalent concentrations of the active components. This phenomenon is attributed to the absence of interacting substances present in crude extracts. For example, extracts were obtained from the fresh herb of *Artemisa annua* L. either by soaking the herb in water followed by wringing out the juice by hand or by pounding the fresh herb to a pulp followed by squeezing out the juice. The extracts were then analyzed for artemisinin concentration and tested against malaria parasites. It was found that the antiplasmodial IC_50_-values were 6–18-fold lower than was expected in terms of their artemisinin content suggesting that the activity of the extracts could not be entirely accounted by their artemisinin content (Wright et al., 2010). Another example are the *Cinchona* alkaloids. There are almost 30 alkaloids described in the bark of *Cinchona officinalis* L. The most well-known of these are quinine, quinidine, and cinchonine and cinchonidine. However, quinine is not the most potent of the alkaloids: quinidine, dihydroquinidine, and cinchonine all have consistently lower 50% inhibitory concentrations (IC_50_) *in vitro*. The combination of quinine with quinidine and cinchonine is 2–10 times more effective *in vitro* against quinine-resistant strains, and the mixture of alkaloids reveals more consistent effects than any of the alkaloids singly used (Druilhe et al., 1988; Karle and Bhattacharjee, 1999). In pharmacokinetic synergy, substances with little or no bioactivity may assist the main active principle to reach the disease target by several mechanisms, e.g., improving bioavailability, or decreasing metabolism and excretion. Comparable effects are not yet known for *C. longa* and needs to be elucidated. In the present investigation, we addressed this question. We have chosen ascorbic acid (vitamin C, ascorbate, C_6_H_12_O_6_) as phytochemical constituent of *C. longa* to investigate the additive or synergistic effects of its combination with curcumin.

Ascorbic acid is a ketolactone and a water-soluble antioxidant. There are two chemical forms of ascorbic acid: the reduced form (ascorbic acid; AA) and the oxidized form (dehydroascorbic acid; DHA; Mamede et al., 2011). AA is well-known for its potent antioxidant properties, as it is able to scavenge free radicals and reactive oxygen species (ROS). Thus, it has been associated with decreased oxidative stress *in vivo* (Carr A. and Frei, 1999; Carr A. C. and Frei, 1999). High doses of AA can reduce inflammatory biomarkers such as C-reactive protein (CRP), tumor necrosis factor (TNF-α), interferon-γ (IFN-γ), and the interleukins IL-1, IL-2, IL-6, IL-8 (Gilliam and St Clair, 2011; Mikirova et al., 2012). AA is a cofactor *in vivo* for enzymes involved in the biosynthesis of collagen, carnitine, neurotransmitters, and neuropeptide hormones as well as enzymes involved in regulation of epigenetic or transcription factors (Rebouche, 1991; Du et al., 2012). Furthermore, it is a cofactor in the synthesis of the neurotransmitters norepinephrine, dopamine, and serotonin and neuropeptide hormones such as oxytocin (Harrison and May, 2009; May et al., 2012). Extensive studies have been carried out on AA in the treatment of cancer and vast literature exists on AA and cancer. In 1949, AA was first proposed to be used for cancer therapy (Klenner, 1949; Mc, 1952). The first comprehensive review on AA and cancer was published in 1979 and an updated review 25 years later (Cameron et al., 1979; González et al., 2005). AA may act as a prodrug causing the formation of AA radical and hydrogen peroxide in the extracellular space (Chen et al., 2007). In a clinical trial, AA exerted antitumor activity in patients with advanced cancer as a stand-alone therapy as well as in combination with other anticancer agents (Hoffer et al., 2008). The conjugation of AA with extracts of medical herbs stimulated apoptosis and disrupted the cell cycle in different cancer cell lines. Furthermore, AA was pro-oxidant generating hydrogen peroxide-dependent cytotoxicity toward various cancer cells without adversely affecting normal cells. AA together with sodium nitrite induced genotoxicity due to oxidative DNA damage. High concentrations of AA killed tumor cells *in vitro* with high efficiency and inhibited angiogenesis in mice bearing sarcoma (Chen et al., 2008; Kuroiwa et al., 2008; Verrax and Calderon, 2009; Yeom et al., 2009; Rozanova Torshina et al., 2010).

In this paper, we have chosen AA because it is widely distributed in many plants and it is also synthetically available. This allows us to investigate the cytotoxicity of the combination of curcumin and AA outside of the plant at exactly defined conditions to clarify their pharmacological effects in the combination. Our strategy was to investigate the combination by isobologram analysis and to investigate the genes which are related to the cytotoxicity induced by curcumin and AA in a broad spectrum of cancers. We postulate that the identification of genes that are specifically regulated by AA and curcumin could improve the understanding of the efficacy of *C. longa* in cancer treatment. We have systematically studied the microarray-based mRNA expression of genes, which influence the cellular response to curcumin and AA in the tumor cell line panel of the National Cancer Institute (NCI), USA to find possible mechanistic explanations for the interaction of these two compounds.

## Materials and methods

### Chemicals

All chemicals were of analytical grade. Curcumin, AA and DMSO were purchased from Sigma-Aldrich (Sigma-Aldrich Corp., St. Louis, MO, USA).

### COMPARE and hierarchical cluster analyses of microarray data

The cancer cell lines of the Developmental Therapeutics Program of NCI consisted of a series of non-small cell lung cancer, colon cancer, renal cancer, ovarian cancer cells, leukemia, melanoma, prostate carcinoma, breast cancer, and tumor cells of the central nervous system. Their origin and processing have been previously reported (Alley et al., 1988). The cytotoxicity induced by AA, curcumin, and standard anticancer drugs of the NCI cell line panel was measured by the sulforhodamine B assay. The 50% inhibition concentrations calculated from dose-response curves and converted to logarithmic values [log_10_IC_50_ (M)] have been deposited at the NCI database (http://dtp.cancer.gov/databases_tools/default.htm). The mRNA microarray hybridization of the NCI cell lines has been reported and deposited at the NCI website (http://dtp.cancer.gov/databases_tools/default.htm). COMPARE analyses were performed to produce rank ordered lists of genes expressed in the NCI cell lines. The methodology has been previously described in detail as a tool to identify candidate genes for drug resistance and sensitivity. To derive COMPARE rankings, a scale index of correlation coefficients (*R*-values) was created from log_10_IC_50_ (M) values of test compounds and microarray-based mRNA expression values. Greater mRNA expression correlated with enhanced drug resistance in the standard COMPARE approach, whereas greater mRNA expression in cell lines indicated drug sensitivity in reverse COMPARE analyses. Pearson's correlation test was used to calculate significance values and rank correlation coefficients as a relative measure for the linear dependency of two variables.

For hierarchical cluster analysis, objects were classified by calculation of distances according to the closeness of between individual distances by means of hierarchical cluster analysis. All objects were assembled into cluster trees (dendrograms). Merging of objects with similar features leads to cluster formation, where the length of the branch indicates the degree of relation. Distances of subordinate cluster branches to superior cluster branches serve as criteria for the closeness of clusters. Thus, objects with tightly related features were clustered closely together, while separation of objects in the dendrogram increased with progressive dissimilarity. Hierarchical clustering and heat-map analysis were performed using Euclidean distance and ward method implemented in “dist,” “hclust,” and “heatmap” functions in R programming (Eisen et al., 1998; Gu et al., 2016). The results were further confirmed using the CIM miner software by use of the one matrix clustered image map (CIM) https://discover.nci.nih.gov/cimminer/oneMatrix.do.

### Cell culture

Drug sensitive CCRF-CEM and multidrug-resistant P-glycoprotein overexpressing CEM/ADR5000 leukemic cells were generously provided by Prof. Axel Sauerbrey (Department of Pediatrics, University of Jena, Jena, Germany). They were cultured in RPMI-1640 medium supplemented with 10% FBS and 1% penicillin/streptomycin (Invitrogen, Darmstadt, Germany). Doxorubicin (5000 ng/mL) was added to maintain overexpression of P-gp (*MDR1, ABCB1*) in resistant cells (Kimmig et al., 1990). Human wild-type HCT116 colon cancer cells (p53^+/+^) and knockout clones (p53^−/−^) derived by homologous recombination (Waldman et al., 1995; Bunz et al., 1998) were generously provided by Dr. B. Vogelstein and H. Hermeking (Howard Hughes Medical Institute, Baltimore, MD, USA). Both colon cancer cells were cultured in DMEM medium supplemented with 10% FBS and 1% penicillin/streptomycin (Invitrogen). Wild-type human U87MG glioblastoma multiform cells and cells transfected with control mock vector or an expression vector harboring *EGFR* cDNA with a deletion in exons 2–7 (U87MG.ΔEGFR), were kindly provided by Dr. W. K. Cavenee (Ludwig Institute for Cancer Research, San Diego, CA, USA; Huang et al., 1997).

### Cell viability assay

Cell viability was evaluated by resazurin assay. This test is based on reduction of the indicator dye, resazurin, to the highly fluorescent resorufin by viable cells. Nonviable cells rapidly lose the metabolic capacity to reduce resazurin and thus produce no fluorescent signal. Glioblastoma and colon cancer cells were harvested with 0.25% trypsin/EDTA (Invitrogen, Germany) and diluted to a final concentration 5 × 10^4^ cells/mL. One hundred microliters of the cell suspension were sowed into the wells of a 96-well-culture plate 1 day before treatment. However, for the leukemia cell lines 2 × 10^4^ cells were sowed in a 96-well-culture plate in a total volume of 100 μL for each well and then the cells were immediately treated. Marginal wells were filled with 200 μL of pure medium, in order to minimize effects of evaporation. Besides, wells filled with medium served as the negative control to determine background fluorescence that may be present. Then, cells were treated with different concentrations of curcumin, vitamin C alone, or combined. After 72 h, 20 μL resazurin (Sigma-Aldrich, Germany) 0.01% w/v in ddH_2_O was added to each well and the plates were incubated at 37°C for 4 h. Fluorescence was measured on an Infinite M2000 Proplate reader (Tecan, Germany) using an excitation wavelength of 544 nm and an emission wavelength of 590 nm. Each assay was done at least two times, with six replicates each. The cytotoxic effect of the treatment was determined as percentage of viability and compared to untreated cells. The calculated cell viability (y-axis) was plotted against the log drug concentration (x-axis) using Microsoft Excel. The obtained curve was used to determine the IC_50_-value, which represented the concentration of the test compound required to inhibit 50% of cell proliferation.

### Statistical analysis

The Loewe additivity model was used to calculate synergetic drug interactions between curcumin and vitamin C in inhibiting cell growth. In this model, the combination index (CI) was defined as CI = (d1/D1)/(d2/D2), where D1 and D2 were the doses of drug 1 and drug 2 that produced an response Y (e.g., 50% inhibition of CCRF-CEM growth) when used alone, d1 and d2 were the doses of drug 1 and drug 2 in combination, which can generate the same response Y. If the CI is equal, less than or more than 1, the combination dose (d1, d2) is termed as additive, synergistic, or antagonistic, respectively. The drug interaction was illustrated geometrically as isobologram.

Pearson's correlation test was used to calculate significance values and rank correlation coefficients as a relative measure for the linear dependency of two variables. This test was implemented into the WinSTAT Program (Kalmia Co.). Pearson's correlation test determined the correlation of rank positions of values. Ordinal or metric scaling of data is suited for the test and transformed into rank positions. There is no condition regarding normal distribution of the data set for the performance of this test. We used Pearson's correlation test to correlate microarray-based mRNA expression of candidate genes with the IC_50_-values for curcumin and ascorbic acid.

The χ^2^-test was applied to bivariate frequency distributions of pairs of nominal scaled variables. It was used to calculate significance values (*p*-values) and rank correlation coefficients (*R*-values) as a relative measure for the linear dependency of two variables. This test was implemented into the WinSTAT program (Kalmia Co.). The χ^2^-test determines the difference between each observed and theoretical frequency for each possible outcome, squaring them, dividing each by the theoretical frequency, and taking the sum of the results. Performing the χ^2^-test necessitated to define cell lines as being sensitive or resistant to curcumin and ascorbic acid. This has been done by taking the median IC_50_-value (log_10_ = −5.1 M) for curcumin and IC_50_-value (log_10_ = −2.7 M) for ascorbic acid as a cut-off threshold.

## Results

### Chemoprofiling of different *Curcuma* species

**Figure 1:**
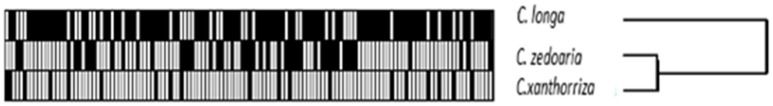
**Dendrogram obtained by hierarchical cluster analysis of phytochemical constituents of *Curcuma longa*, *C*. *zedoaria*, and *C. xanthorrhiza***. The chemical compounds included in this cluster analysis are listed in detail in Supplementary Table 1.

As a first step, we established chemoprofiles of three *Curcuma* species (*C. longa, C. zedoaria*, and *C. xanthorrhiza*) based on the chemical compositions of these species deposited at Dr. Duke's Phytochemical and Ethnobotanical Databases (http://www.arsgrin.gov/cgi-bin/duke/farmacy2.pl). We subjected the chemical composition of these plants to hierarchical cluster analysis (Figure 1). A total of 114 phytochemicals have been included in the analysis, which are listed in detail in Supplementary Table 1. Three compounds were commonly found in all three *Curcuma* species (curcumin, D-camphor, and desmethoxycurcumin). Ten compounds were found in two of the three species, whereas all other compounds were found in only one *Curcuma* species. This specific distribution of phytochemicals enables specific clustering and separation of the *Curcuma* species.

### Cytotoxicity of AA in the NCI panel of cell lines

**Figure 2:**
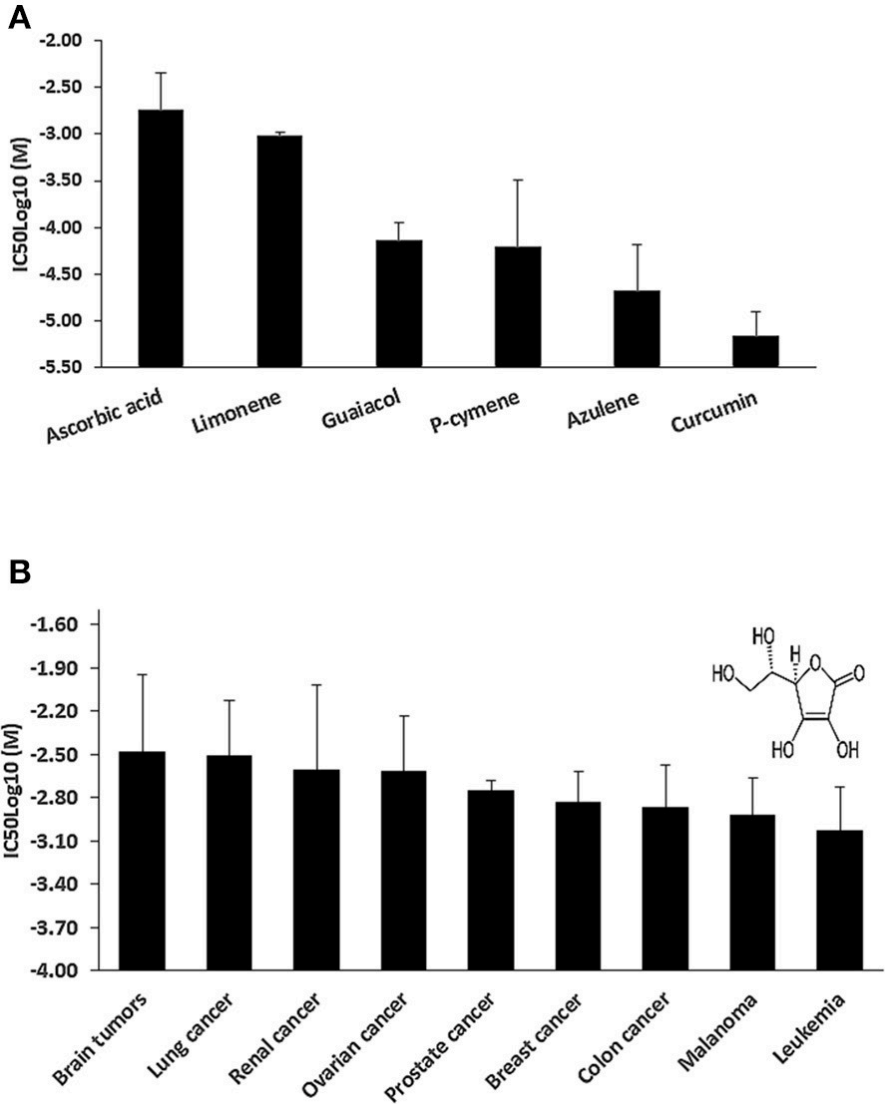
**(A)** Mean IC_50_log_10_-values of selected cytotoxic phytochemicals from *Curcuma longa* for the NCl tumor cell line panel as assayed by the sulforhodamine B-test. **(B)** Tumor-type-dependent cytotoxicity of ascorbic acid. Insert, chemical structure of ascorbic acid.

We hypothesized that the cytotoxic effect of *C. longa* against cancer cells is not solely caused by its main compound, curcumin, but that other compounds may also contribute to this activity of the plant. To prove this hypothesis, we mined the NCI database for compounds found in *C. longa* and six compounds were identified (Figure 2A), i.e., AA, limonene, guaiacol, p-cymene, azulene, and curcumin. Although AA was not the most toxic compound among the six tested, we decided to continue our investigations with AA, because of its far distribution not only in *C. longa* but also in many other plants as well and it's enormous relevance for human health in general. Further, investigations were then carried out using AA in the NCI panel of cell lines. Leukemia and melanoma cell lines were most sensitive, while brain and lung cancer cell lines were the most resistant ones (Figure 2B). Established anticancer drugs frequently show high sensitivity toward leukemia, but resistance toward melanoma. Hence, it is interesting that AA was active against melanoma cell lines.

### Cytotoxicity of curcumin and AA toward drug resistant cancer cell lines

Drug-resistant cell lines with different resistance mechanisms (P-glycoprotein, EGFR, mutant p53) toward curcumin and AA were determined. All the cell lines were treated with varying concentrations of curcumin and AA for 72 h, their growth was inhibited in a dose-dependent manner, albeit at different efficacy. The IC_50_-values were calculated from the dose response curves and summarized in Table 1. Curcumin inhibited cell growth at lower concentrations than AA. The range of IC_50_-values was 5.22–58.3 μM for curcumin, while that of AA was 389.1–561.2 μM. The CCRF-CEM and CEM/ADR5000 leukemia cell lines were inhibited at concentrations of 5.22 and 6.33 μM, respectively. The U87MG.ΔEGFR-transfectant glioblastoma cells exhibited sensitivity toward curcumin with an IC_50_-value of 46.1 μM, which was slightly lower than the IC_50_-value of wild-type U87MG cells 49.6 μM. Interestingly, the HCT166p53^−/−^ colon cancer cell line was preferentially inhibited by AA with an IC_50_-value of 506.9 μM compared to HCT116p53^+/+^ wild-type cells 561.2 μM. The degrees of resistance were calculated by dividing the IC_50_ of the resistant cell line by the IC_50_ of the sensitive cell line. Compared to the high degrees of resistance of these drug-resistant cell lines to standard drugs such as doxorubicin (Hall et al., 2009), curcumin and AA inhibited these cell lines with similar efficacies. The degrees of resistance were in a range of 0.9–1.41 (Table 1).

**Table 1:** **IC_50_-values of curcumin and vitamin C toward various cell lines**.

**Cell lines**	**Curcumin**		**Vitamin C**	
	**IC_50_ (μM)**	**Degree of resistance**	**IC_50_ (μM)**	**Degree of resistance**
CCRF-CEM	5.22 ± 0.15		502.61 ± 2.24	
CEM/ADR5000	6.33 ± 0.07	1.21	523.12 ± 2.19	1.04
U87MG	49.60 ± 16.40		389.10 ± 53.50	
U87MG.ΔEGFR	46.10 ± 4.80	0.92	532.10 ± 76.40	1.37
HCT116p^+/+^	41.20 ± 12.06		561.20 ± 58.80	
HCT116p53^−/−^	58.30 ± 4.30	1.41	506.90 ± 20.40	0.90

*Shown are mean values ± SD of four independent experiments with each six parallel measurements. Degrees of resistance were calculated by the IC_50_ resistant cell line/IC_50_ sensitive cell line ratio*.

### Cytotoxic effects of combination treatments of curcumin and AA

**Figure 3:**
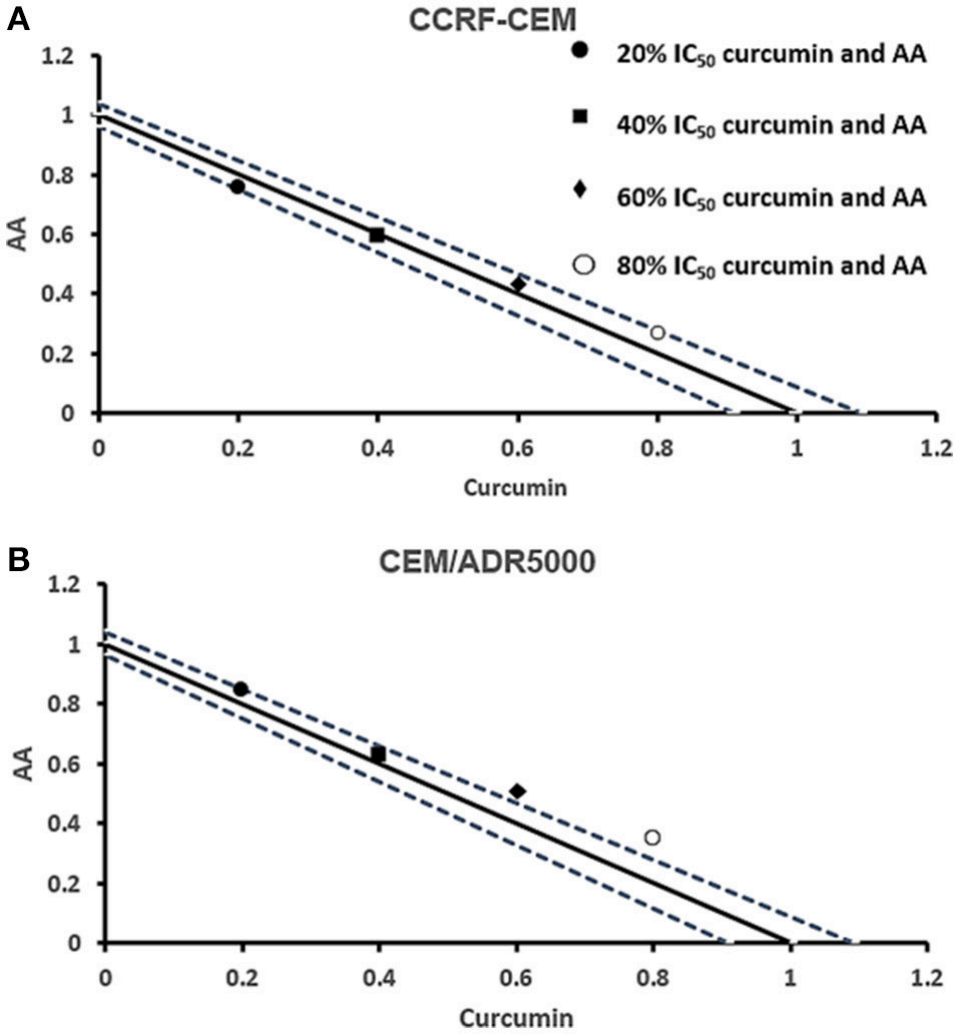
**Isobologram analysis for the interaction of various combinations of curcumin and ascorbic acid on (A)** CCRF-CEM and **(B)** CEM/ADR5000 leukemia cell lines.

**Figure 4:**
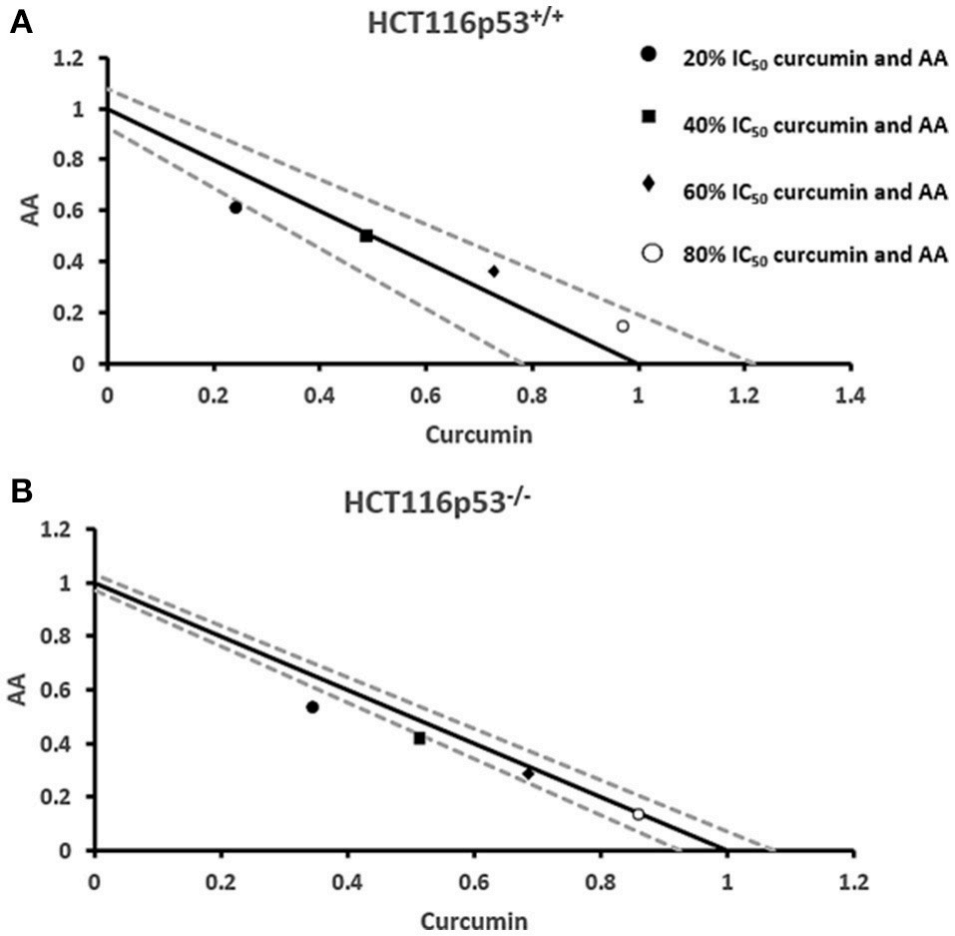
**Isobologram analysis for the interaction of various combinations of curcumin and ascorbic acid on (A)** HCT116p53^+/+^ and **(B)** HCT116p53^−/−^ colon cancer cell lines.

**Figure 5:**
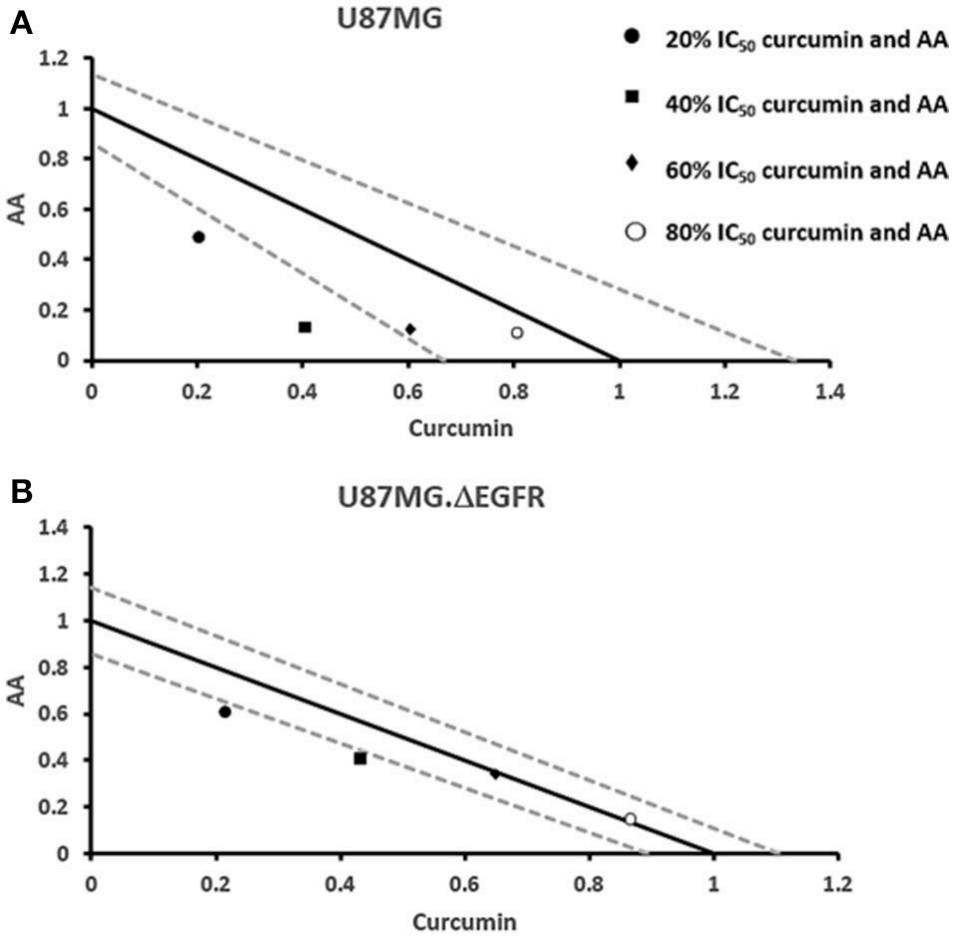
**Isobologram analysis for the interaction of various combinations of curcumin and ascorbic acid on (A)** U87MG and **(B)** U87MG.ΔEGFR glioblastoma cell lines.

Next, we addressed the question, whether the combination of curcumin and AA exhibits additive or synergistic growth inhibition of cancer cells. We applied a universal reference model for evaluating the effects of drug interaction, i.e., the Loewe additivity model (isobologram analysis). The cancer cell lines were treated with varying concentrations of AA at indicated concentrations of curcumin for 72 h. In CCRF-CEM and CEM/ADR5000 cells, the IC_50_-values of curcumin in combination with AA were reduced by less than half of the IC_50_ of curcumin alone. In the glioblastoma and colon cancer cell lines, the IC_50_-value of two of the curcumin concentrations (20% IC_50_ curcumin and 40% IC_50_ curcumin) decreased with increasing AA concentrations less than the IC_50_ of curcumin alone. However, the two other concentrations of curcumin reduced the IC_50_ in combination with AA by less than half of the IC_50_-value of curcumin alone. Dose-normalized IC_50_ isobolograms for all cell lines were generated by plotting the combination treatment IC_50_-values of curcumin against AA. Additive effects were observed in CCRF-CEM and CEM/ADR5000 (Figure 3) as well as in HCT116p53^+/+^ and HCT116p53^−/−^ cell lines (Figure 4), whereas supra/additive effects were visible in U87MG and U87MG.ΔEGFR cells (Figure 5).

### COMPARE and hierarchical cluster analyses of mRNA expressions

**Figure 6:**
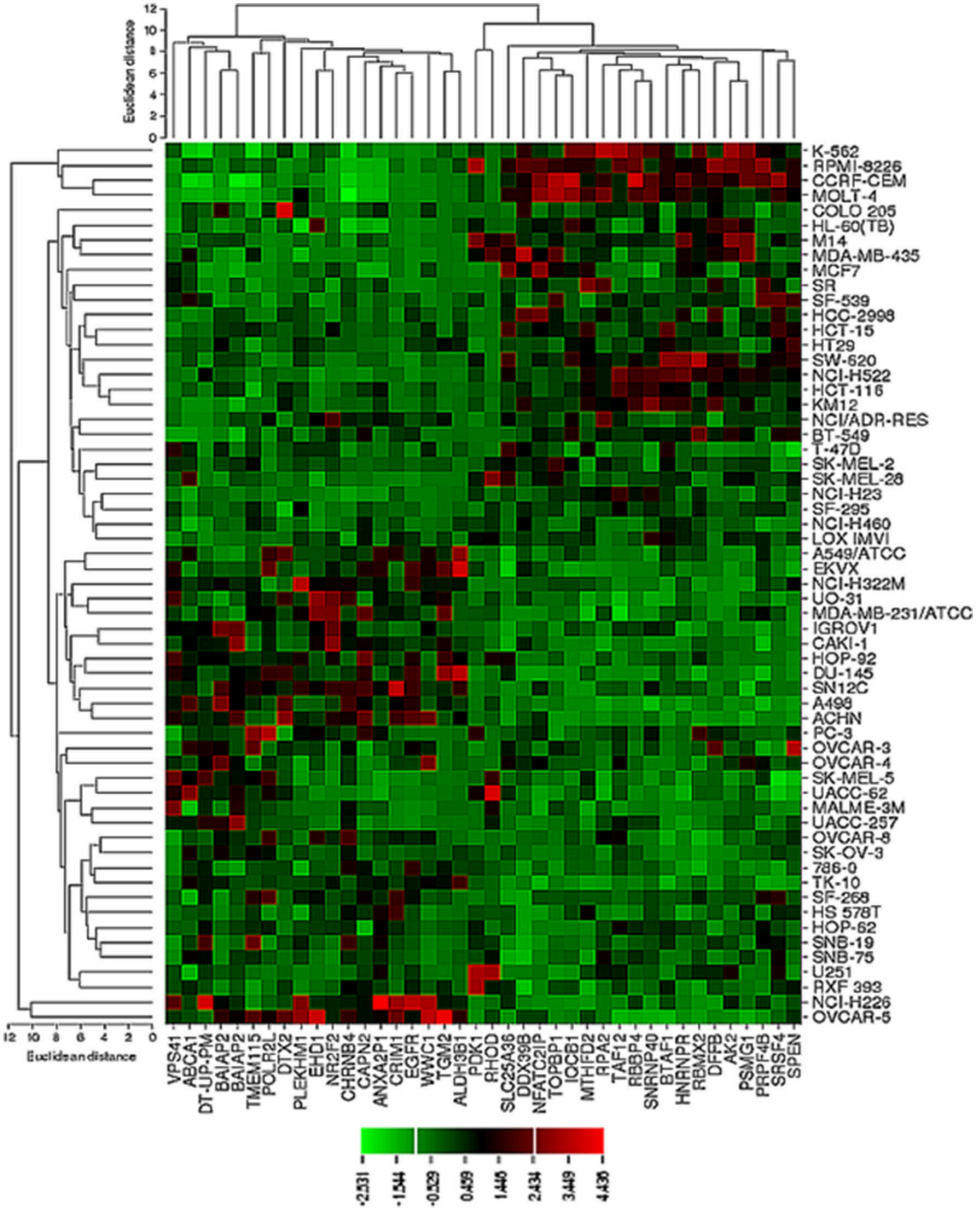
**Dendrograms and cluster image map of curcumin obtained by hierarchical cluster analysis of mRNA expression of 40 genes in the NCI cell line panel as analyzed by the Novartis microarray platform**. The dendrogram on the left shows the clustering of cell lines and the dendrogram on the top shows the clustering of genes. The cluster image map shows each single mRNA expression value obtained by microarray analysis. The expression values have been normalized and color-coded.

**Figure 7:**
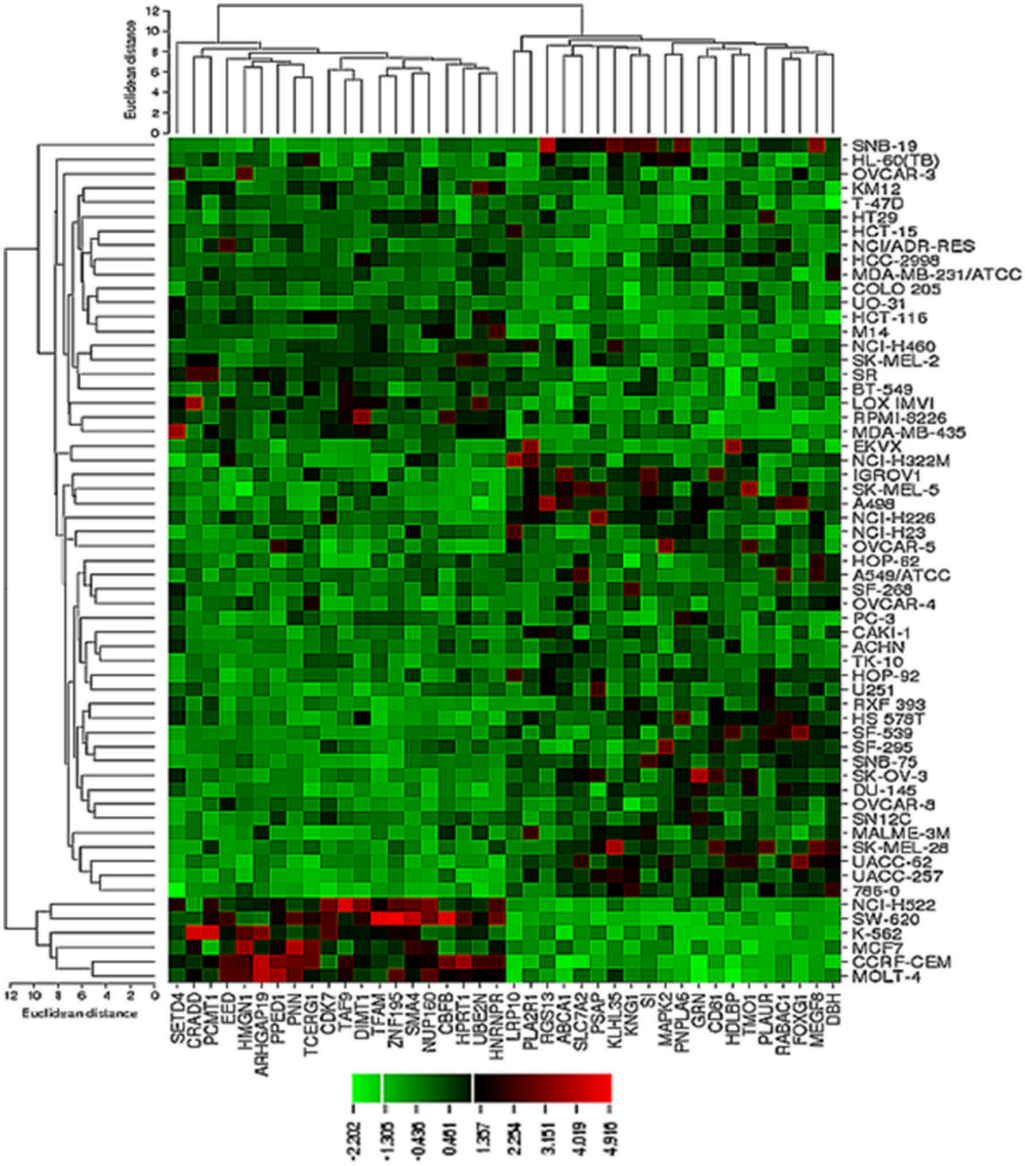
**Dendrograms and cluster image map of vitamin C obtained by hierarchical cluster analysis of mRNA expression of 40 genes in the NCI cell line panel as analyzed by the Novartis microarray platform**. The dendrogram on the left shows the clustering of cell lines and the dendrogram on the top shows the clustering of genes. The cluster image map shows each single mRNA expression value obtained by microarray analysis. The expression values have been normalized and color-coded.

The transcriptome-wide mRNA expression of the NCI cell lines based on the Novartis microarray platform was investigated by COMPARE analyses and correlated to the log_10_IC_50_ (M) values for curcumin and AA. This bioinformatical approach was performed to identify novel putative factors associated with cellular response to curcumin and AA. The top 20 genes with direct and top 20 genes with inverse correlation co-efficient are shown in Tables 2, 3. These genes were subjected to hierarchical cluster analysis to analyze, whether the expression profiles of these genes may predict sensitivity or resistance of the cells to curcumin and AA. The mRNA expression of the identified genes were subjected to hierarchical cluster analysis and cluster image mapping (Figures 6, 7). The resulting dendogram with the cell lines analyzed on the left can be divided into five major clusters for curcumin and four clusters for ascorbic acid. Using the chi-square test, we analyzed whether the distribution of cell lines being sensitive or resistant to curcumin and AA was statistically significant. As shown in Table 4, the distribution of sensitive or resistant cell lines on the dendogram was significantly different indicating that cellular response to curcumin or AA was predictable by the mRNA expression of these genes. Therefore, it is interesting to know the function of these genes. The specific functions of the proteins encoded by the genes were diverse and included signal transduction, transcription factors, proteasome deregulation, apoptosis regulating genes, proliferation-related genes, pro- as well as anti-oxidative genes (Tables 2, 3). AA induced genes that belonged to the functional groups of transcription factors (*TFAM, TCERG1, RGS13*, and *C11orf31*), apoptosis-regulating genes (*CRADD, CDK7, CDK19, CD81, TOM1*) and signal transduction genes (*NR1D2, HMGN1, ABCA1, DE4ND4B, TRIM27*). Curcumin induction resulted in DNA repair genes (*TOPBP1, RPA2*), mRNA metabolism genes (*RBBP4, HNRNPR, SRSF4, NR2F2, PDK1*, and *TGM2*), signal transduction genes *(WWC1, DTX2, EGFR, CHRNB4, VPS41, CRIM1*), proliferation-related genes (*RHOD*), apoptosis-regulating genes *(DFFB*), and transporter genes (*ABCA1*).

**Table 2:** **Meta-data of genes shown in the cluster analysis whose mRNA expression correlated with the log_10_IC_50_ values of curcumin in the NCI tumor cell line panel**.

**Gene symbol**	**Gene acc no**.	**Gene name**	**Gene function**
*ABCA1*	AI344681	ATP-binding cassette, sub-family A (ABC1), member 1	cAMP-dependent and sulfonylurea-sensitive anion transporter. Key gatekeeper influencing intracellular cholesterol transport.
*AK2*	U54645	Adenylate kinase 2	Catalyzes the reversible transfer of the terminal phosphate group between ATP and AMP.
*ALDH3B1*	U10868	Aldehyde dehydrogenase 3 family, member B1	Oxidizes medium and long chain saturated and unsaturated aldehydes. Protective role against the cytotoxicity induced by lipid peroxidation.
*ANXA2P1*	M62896	Annexin A2 pseudogene 1	Involved in cell proliferation and membrane physiology and is related to cancer progression.
*BAIAP2*	AB015020	BAI1-associated protein 2	Adapter protein that links membrane-bound small G-proteins to cytoplasmic effector proteins.
*BAIAP2*	AB015019	BAI1-associated protein 2	Adapter protein that links membrane-bound small G-proteins to cytoplasmic effector proteins. Participates in actin bundling, if associated with EPS8. Promoting filopodial protrusions.
*BTFA1*	AF038362	B-TFIID transcription factor-associated, 170kDa	Regulates transcription in association with TATA binding protein (TBP). Removes TBP from the TATA box.
*CAPN2*	M23254	Calpain 2 (m/II) large subunit	Calcium-regulated non-lysosomal thiol-protease, which catalyzes proteolysis of substrates involved in cytoskeletal remodeling and signal transduction.
*CHRNB4*	U48861	Cholinergic receptor, nicotinic, beta 4	Leads to opening of an ion-conducting channel across the plasma membrane.
*CRIM1*	AI651806	Cysteine rich transmembrane BMP regulator 1 (chordin-like)	Role in capillary formation and maintenance during angiogenesis.
*DDX39B*	AC002400	DEAD (Asp-Glu-Ala-Asp) box polypeptide 39B	Involved in nuclear export of spliced and unspliced mRNA. Assembling component of the TREX complex. Has both RNA-stimulated ATP binding/hydrolysis activity and ATP-dependent RNA unwinding activity.
*DFFB*	AF064019	DNA fragmentation factor, 40kDa, beta polypeptide	Induces DNA fragmentation and chromatin condensation during apoptosis.
*DT-UP-PM*	U38980	DTX2P1-UPK3BP1-PMS2P11	Encodes a putative E3-ubiquitin ligase with no known biological function.
*DTX2*	AI138834	Deltex 2	Regulator of Notch signaling.
*EGFR*	X00588	Epidermal growth factor receptor	Receptor tyrosine kinase activating several signaling cascades. Activates the NF-kappa-B signaling cascade.
*EHD1*	AF001434	EH-domain containing 1	Acts in early endocytic membrane fusion and membrane trafficking of recycling endosomes.
*HNRNPR*	AF000364	Heterogeneous nuclear ribonucleoprotein R	Component of ribonucleosomes, which are complexes of >20 other heterogeneous nuclear ribonucleoproteins (hnRNP). hnRNP play an important role in processing of precursor mRNA in the nucleus.
*IQCB1*	D25278	IQ motif containing B1	Involved in ciliogenesis.
*MTHFD2*	X16396	Methylenetetrahydrofolate dehydrogenase (NADP+ dependent) 2	Encodes a nuclear-encoded mitochondrial bifunctional enzyme with methylenetetrahydrofolate dehydrogenase and methenyltetrahydrofolate cyclohydrolase activities.
*NFATC2IP*	AA152202	Nuclear factor of activated T-cells, cytoplasmic, calcineurin-dependent 2 interacting protein	In T-helper 2 (Th2) cells, regulates NFAT-driven transcription of cytokine genes IL3, IL4, IL5 and IL13. Recruits PRMT1 to the IL4 promoter and facilitates subsequent histone acetylation at the IL4 locus. Promotes robust cytokine expression. Down-regulates formation of poly-SUMO chains by UBE2I/UBC9.
*NR2F2*	M64497	Nuclear receptor subfamily 2	Ligand-activated transcription factor. Activated by high concentrations of 9-cis-retinoic acid and all-trans-retinoic acid, but not by dexamethasone, cortisol or progesterone (*in vitro*). Regulation of the apolipoprotein A-I gene transcription.
*PDK1*	L42450	Pyruvate dehydrogenase kinase, isozyme 1	Role in regulation of glucose and fatty acid metabolism and homeostasis via phosphorylation of the pyruvate dehydrogenase subunits PDHA1 and PDHA2. Role in cellular responses to hypoxia. Important for cell proliferation under hypoxia.
*PLEKHM1*	AB002354	Pleckstrin homology domain containing, family M (with RUN domain) member 1	Involved in vesicular transport in the osteoclast. Role in sialyl-lex-mediated transduction of apoptotic signals.
*POLR2L*	N24355	Polymerase (RNA) II (DNA directed) polypeptide L	DNA-dependent RNA polymerase catalyzes the transcription of DNA into RNA using the four ribonucleoside triphosphates as substrates.
*PRPF4B*	U48736	Pre-mRNA processing factor 4B	Role in pre-mRNA splicing. Phosphorylates SF2/ASF.
*PSMG1*	AJ006291	Proteasome (prosome, macropain) assembly chaperone 1	Chaperone protein, which promotes assembly of the 20S proteasome as part of a heterodimer with PSMG2.
*RBBP4*	X74262	Retinoblastoma binding protein 4	Core histone-binding subunit that may target chromatin assembly factors, chromatin remodeling factors and histone deacetylases to their histone substrates.
*RBMX2*	AL050405	RNA binding motif protein, X-linked 2	Encodes RNA binding motif protein, X-linked 2.
*RHOD*	U61374	Ras homolog family member D	Involved in endosome dynamics. Coordinates membrane transport with the function of the cytoskeleton.
*RPA2*	J05249	Replication protein A2	Required for DNA recombination, repair and replication. Required for the recruitment of the DNA double-strand break repair factor RAD51 to chromatin in response to DNA damage.
*SLC25A36*	AL049246	Solute carrier family 25 (pyrimidine nucleotide carrier), member 36	To catalyze uptake of pyrimidine (deoxy) nucleotide triphosphates into the mitochondrial matrix in exchange for internal pyrimidine (deoxy) nucleotide monophosphates or (deoxy) nucleotide diphosphates.
*SNRNP40*	AF090988	Small nuclear ribonucleoprotein 40kDa	Component of the U5 small nuclear ribonucleoprotein (snRNP) complex.
*SPEN*	AL096858	*Spen* family transcriptional repressor	Serve as nuclear matrix platform that organizes transcriptional responses. Essential corepressor protein, which regulates different key pathways such as the Notch pathway. Represses transcription via the recruitment of large complexes containing histone deacetylase proteins.
*SRSF4*	LI4076	Serine/arginine-rich splicing factor 4	Role in alternative splice site selection during pre-mRNA splicing. Represses the splicing of MAPT/Tau exon 10.
*TAF12*	X84002	TATA box binding protein (TBP)-associated factor	TAFs are components of the transcription factor IID (TFIID) complex, PCAF histone acetylase complex and TBP-free TAFII complex (TFTC). TAFs components-TIIFD are essential for mediating regulation of RNA polymerase transcription.
*TGM2*	M55153	Transglutaminase 2	Catalyzes the cross-linking of proteins and conjugation of polyamines to proteins.
*TMEM115*	U09584	Transmembrane protein 115	Role in retrograde transport of proteins from the Golgi to the endoplasmic reticulum.
*TOPBP1*	D87448	Topoisomerase (DNA) II binding protein 1	Required for DNA replication. Down-regulates E2F1 activity and inhibits E2F1-dependent apoptosis during G1/S transition and after DNA damage.
*VPS41*	U87309	Vacuolar protein sorting 41	Required for vacuolar assembly and vacuolar traffic.
*WWC1*	AB020676	WW and C2 domain containing 1	Regulator of the Hippo/SWH (Sav/Wts/Hpo) signaling pathway, which plays a role in tumor suppression by restricting proliferation and promoting apoptosis. Transcriptional coactivator of ESR1. Regulates collagen-stimulated activation of the ERK/MAPK cascade.

*Gene information was taken from the OMIM database, National Cancer Institute, USA (http://www.ncbi.nlm.nih.gov/Omim/), and from the GeneCard database of the Weizman Institute of Science, Rehovot, Israel (http://bioinfo.weizmann.ac.il/cards/index.html)*.

**Table 3:** **Meta-data of genes shown in the cluster analysis of whose mRNA expression correlated with log_10_IC_50_-values of vitamin C in the NCI tumor cell line panel**.

**Gene symbol**	**GenBank acc no**.	**Gene name**	**Gene function**
*ABCA1*	A1344681	ATP-binding cassette, sub-family A (ABC1), member 1	cAMP-dependent and sulfonylurea-sensitive anion transporter. Key gatekeeper influencing intracellular cholesterol transport.
*ARHGAP19*	U79256	Rho GTPase activating protein 19	GTPase activator for Rho-type GTPases.
*CBFB*	L20298	Core-binding factor, beta subunit	CBF binds to enhancers and promoters, including murine leukemia virus, polyomavirus enhancer, T-cell receptor enhancers, and LCK, IL3, and GM-CSF promoters.
*CD81*	M33680	CD81	Regulation of lymphoma cell growth. Involved in signal transduction.
*CDK7*	X77743	Cyclin-dependent kinase 7	The catalytic subunit of the CDK-activating kinase (CAK) complex.
*CRADD*	U84388	CASP2 and RIPK1 domain containing adaptor with death domain	Apoptotic adaptor molecule specific for caspase-2 and FASL/TNF receptor-interacting protein RIP.
*DBH*	X13255	Dopamine beta-hydroxylase (dopamine beta-monooxygenase)	Conversion of dopamine to noradrenaline.
*DIMT1*	AF091078	DIM1 dimethyladenosine transferase	Dimethylates two adjacent adenosines in the loop of a conserved hairpin near the 3′-end of 18S rRNA in the 40S particle
*EED*	AF080227	Embryonic ectoderm development	Transcriptional repression of target genes. Constituent of a recruiting platform for DNA methyltransferases, thereby involved in epigenetic repression.
*FOXG1*	X74143	Forkhead box G1	Transcription repression factor.
*GRN*	AF055008	Granulin	Cytokine-like activity. Role in inflammation, wound repair, and tissue remodeling.
*HDLBP*	M64098	High density lipoprotein binding protein	Role in cell sterol metabolism. Protects cells from over-accumulation of cholesterol.
*HMGN1*	J02621	High mobility group nucleosome binding domain 1	Binds to the inner side of the nucleosomal DNA thus altering the interaction between the DNA and the histone octamer which maintains transcribable genes in a unique chromatin conformation.
*HNRNPR*	AF000364	Heterogeneous nuclear ribonucleoprotein R	Component of ribonucleosomes, which are complexes of >20 other heterogeneous nuclear ribonucleoproteins (hnRNP). hnRNP play an important role in processing of precursor mRNA in the nucleus.
*HPRT1*	M31642	Hypoxanthine phosphoribosyltransferase 1	Transfers 5-phosphoribosyl from 5-phosphoribosylpyrophosphate onto purine. Generation of purine nucleotides through the purine salvage pathway.
*KLHL35*	AA471042	Kelch-like family member 35	A protein coding gene.
*KNG1*	K02566	Kininogen 1	Role in blood coagulation. Inhibits the thrombin- and plasmin-induced aggregation of thrombocytes.
*LRP10*	AL080164	Low density lipoprotein receptor-related protein 10	Receptor involved in the internalization of lipophilic molecules and/or signal transduction. May be involved in the uptake of lipoprotein APOE in liver.
*MAP2K2*	L11285	Mitogen-activated protein kinase kinase 2	Catalyzes the concomitant phosphorylation of threonine and tyrosine residue in a Thr-Glu-Tyr sequence located in MAP kinases. Activates ERK1 and ERK2 MAP kinases.
*MEGF8*	AB011541	Multiple EGF-like-domains 8	Encodes a single pass membrane protein which participates in developmental regulation and cellular communication
*NUP160*	D83781	Nucleoporin 160 kDa	Involved in poly (A)+ RNA transport.
*PCMT1*	D25547	Protein-L-isoaspartate (D-aspartate) O-methyltransferase	Catalyzes methyl esterification of L-isoaspartyl and D-aspartyl residues in peptides and proteins repair and/or degradation of damaged proteins.
*PLA2R1*	U17034	Phospholipase A2 receptor 1	Receptor for secretory phospholipase A2. Activation of the mitogen-activated protein kinase (MAPK) cascade to induce cell proliferation, production of lipid mediators, and selective release of arachidonic acid in bone marrow-derived mast cells. Involved in responses in proinflammatory cytokine productions during endotoxic shock.
*PLAUR*	X74039	Plasminogen activator, urokinase receptor	Role in localizing and promoting plasmin formation. Mediates the proteolysis-independent signal transduction activation effects of uPA.
*PNN*	U77718	Pinin, desmosome associated protein	Transcriptional activator of the E-cadherin gene. Regulation of alternative pre-mRNA splicing. Regulates specific excision of introns in specific transcription subsets. Involved in the establishment and maintenance of epithelia cell-cell adhesion. Potential tumor suppressor for renal cell carcinoma.
*PNPLA6*	AJ004832	Patatin-like phospholipase domain containing 6	Deacylation of intracellular phosphatidylcholine generating glycerophosphocholine.
*PPWD1*	D38552	Peptidylprolyl isomerase domain and WD repeat containing 1	Accelerates the folding of proteins. Involved in pre-mRNA splicing.
*PSAP*	J03077	Prosaposin	Stimulates the hydrolysis of glucosylceramide by beta-glucosylceramidase and galactosylceramide by beta-galactosylceramidase. Behaves as a myelinotrophic and neurotrophic factor.
*RABAC1*	AJ133534	Rab acceptor 1	General Rab protein regulator required for vesicle formation from the Golgi complex. May control vesicle docking and fusion.
*RGS13*	AF030107	Regulator of G-protein signaling 13	Inhibits signal transduction by increasing the GTPase activity of G protein alpha subunits.
*SETD4*	AB004848	SET domain containing 4	Role in protein encoding.
*SI*	X63597	Sucrase-isomaltase (alpha-glucosidase)	Role in the final stage of carbohydrate digestion.
*SLC7A2*	D29990	Solute carrier family 7 (cationic amino acid transporter, y+ system), member 2	Transport of cationic amino acids (arginine, lysine and ornithine). Regulatory role in activation of macrophages.
*SMA4*	X83300	Glucoridase	Required for signal transduction and it also defines a conserved family of transforming growth factor beta pathway components.
*TAF9*	U21858	TATA box binding protein (TBP)-associated factor	Gene regulation associated with apoptosis. Regulation of RNA polymerase II-mediated transcription.
*TCERG1*	AF017789	Transcription elongation regulator 1	Transcription factor that binds RNA polymerase II and inhibits the elongation of transcripts from target promoters in a TATA box-dependent manner.
*TFAM*	M62810	transcription factor A	Mitochondrial transcription regulation.
			Maintenance of normal levels of mitochondrial DNA. Organizing and compacting mitochondrial DNA.
*TOM1*	AJ006973	Target of myb1	Involved in intracellular trafficking. Probable association with membranes.
*UBE2N*	D83004	Ubiquitin-conjugating enzyme E2N	The UBE2V1-UBE2N and UBE2V2-UBE2N heterodimers catalyze the synthesis of non-canonical “Lys-63”-linked polyubiquitin chains. This type of polyubiquitination does not lead to protein degradation by the proteasome. Mediates transcriptional activation of target genes. Plays a role in the control of the cell cycle and differentiation. Plays a role in the error-free DNA repair pathway and contributes to the survival of cells after DNA damage. Induction and expression of NF-kappa-B and MAPK-responsive inflammatory genes.
*ZNF195*	AF003540	Zinc finger protein 195	May be involved in transcriptional regulation.

*Gene information was taken from the OMIM database, National Cancer Institute, USA (http://www.ncbi.nlm.nih.gov/Omim/), and from the GeneCard database of the Weizman Institute of Science, Rehovot, Israel (http://bioinfo.weizmann.ac.il/cards/index.html)*.

**Table 4:** **Separation of clusters of NCI cell lines obtained by hierarchical cluster analyses for curcumin (Figure 6) or ascorbic acid (Figure 7)**.

	**Sensitive**	**Resistant**
**CURCUMIN**		
Partition	<–5.1 M	≥−5.1 M
Cluster1	0	4
Cluster2	5	11
Cluster3	16	3
Cluster4	10	8
Cluster5	1	1
Chi-square test	*P* = 4.59 ^*^ 10^−3^	
**ASCORBIC ACID**		
Partition	<−2.7 M	≥−2.7 M
Cluster 1	2	0
Cluster 2	5	14
Cluster 3	12	20
Cluster 4	0	6
Chi-square	*P* = 0.050	

The median log_10_IC_50_-values (–5.1 M for curcumin and –2.7 M for ascorbic acid) were used as cut-off to separate tumor cell lines as being “sensitive” or “resistant.”

## Discussion

Plants are perhaps earth's most accomplished chemists. They produce thousands of specialized secondary metabolites, and during the evolution of life plants developed multi-targeted chemicals to fulfill diverse tasks. *C. longa* contains some 30 different phytochemicals. In the present study, we analyzed the cytotoxicity of a combination of two of these phytochemicals of *C. longa*, curcumin and AA. If applied alone, curcumin and AA were cytotoxic toward cell lines of different tumor types with curcumin exhibiting stronger cytotoxicity than AA. Our results are in line with other reports on the inhibitory activity of curcumin (Bimonte et al., 2016; Guzzarlamudi et al., 2016; Kasi et al., 2016; Ye et al., 2016; Yu et al., 2016; Zeng et al., 2016) and AA (Chen et al., 2015; Fukui et al., 2015; Jacobs et al., 2015; Sunil Kumar et al., 2015; Venturelli et al., 2015). The anticancer effects of curcumin *in vitro* and *in vivo* are primarily due to the activation of apoptotic pathways in cancer cells as well as the inhibition of mechanisms related to the tumor microenvironments such as inflammation, angiogenesis, invasion, and metastasis. In particular, curcumin targets numerous therapeutically important cancer signaling pathways such as p53, Ras, PI3K, AKT, Wnt, β-catenin, mTOR, and so on.

AA also reveals anticancer activity *in vitro* and *in vivo*, however at higher concentration than curcumin. A number of suggestions have been put forth on the potential mechanisms, by which AA causes death of cancer cells. The most common is that AA is a precursor for hydrogen peroxide (H_2_O_2_) generation, which is considered to be preferentially cytotoxic to cancer cells (Parrow et al., 2013). Exposure to AA concentrations of up to 5 mM for 1 h resulted in decreased survival of cancer cells and cell death was dependent on H_2_O_2_ production mediated by extracellular AA oxidation (Chen et al., 2005). Furthermore, intercellular metals contribute to the production of H_2_O_2_, with AA losing an electron to form a radical molecule. The free electron is donated to a transition metal. This reduced metal is then available to react with molecular oxygen resulting in the generation of H_2_O_2_. In the presence of AA, H_2_O_2_ reacts with another transition metal ion such as ferrous ion to generate a hydroxyl radical (Kehrer, 2000). The tumor suppressor p53 may also play a role for this activity. P53-positive cell lines were more sensitive to both AA and H_2_O_2_ treatment than p53-deficient ones (Kim et al., 2012).

Thus, having two compounds from one plant with diverse mechanisms of action, we were interested to evaluate, whether their combination would reveal synergistic, antagonistic, or additive interactions. Understanding drug-drug interactions always represents a critical issue in the drug development process, since clinically relevant changes in exposure of co-administered drugs can lead to reduced efficacy or, conversely, adverse drug reactions, depending on the therapeutic window of the drugs. The latter becomes especially important with anti-cancer medications, since they are typically administered at or close to the maximally tolerated dose (Waters, 2015). In this study, we applied isobologram analyses to evaluate the nature of interaction of curcumin and AA. Isobologram analysis is considered as gold standard to provide evidence for drug interactions. The combination of curcumin and AA exhibited additive effects in leukemia and colon cancer cell lines and supra-additive effects in the glioblastoma cell lines. Our hypothesis is that both natural products may work together by different molecular pathways to achieve their overall cytotoxicity. Unlike chemically synthesized drugs, natural products might be active at lower doses and over longer periods of incubation, which could further support the appearance of additive effects.

We further analyzed molecular determinants of sensitivity and resistance of cancer tumor cell lines toward curcumin and AA. We correlated the IC_50_-values expressed on induction by curcumin and AA of 60 tumor cell lines by COMPARE analysis of microarray-based transcriptome-wide mRNA expression levels of these cell lines (Scherf et al., 2000). We identified genes from diverse functional groups, which were associated with response of the tumor cells toward curcumin and AA. Under curcumin treatment, these groups of genes included DNA repair, mRNA metabolism, signal transduction, angiogenesis, proliferation, apoptosis etc. While for AA treatment, the COMPARE analysis provided genes that are involved in signal transduction, transcription factors, and apoptosis. Although the exact function of these genes for cellular responsiveness to curcumin or AA treatment is still unknown, we have some clues of explanation. On treatment with AA the following genes were downregulated: *SETD4, TAF9, PNN, CDK7, TFAM*, and *PPWD1*. *SETD4* is a methyltransferase, which is involved in carcinogenesis. Its down-regulation suppressed cellular proliferation and delayed the G1/S cell cycle transition without affecting apoptosis. Furthermore, its knockdown decreased cyclin D1 (Faria et al., 2013). The TATA-binding protein associated factor 9 (TAF9) interacts with oncogenic GLI family members to form GLI-TAF9 binding, which is important for carcinogenesis activity and malignant growth (Yoon et al., 2015). PNN is a nuclear and cell adhesion-related protein participating in the regulation of gene expression and thereby, positively promoting cell-cell adhesion, and negatively affecting cell migration and cell proliferation (Shi et al., 2001). Cyclin-dependent kinase 7 (CDK7), which promotes transcription during the cell cycle, is critical for the survival of cancer cells. The inhibition of CDK7 suppressed proliferation and induced apoptotic cell death (Wang et al., 2015). Mitochondrial transcription factor A (TFAM), a member of the high mobility group (HMG) box protein family, is required for mitochondrial DNA replication and transcription. HMG proteins are often overexpressed in cancer cells and are involved in apoptosis regulation (Krynetskaia et al., 2009; Vander Heiden et al., 2009). TFAM may play a significant role in tumorigenesis (Guo et al., 2011). PPWD1 has a well-characterized peptide domain (WD40 domain for PPWD1), and this domain has also a critical role in carcinogenesis. The WD40 domain mediates signal transduction and transcriptional regulation during cell cycle and apoptosis. PPWD1 may serve as target for drug development (Davis et al., 2008; Jeon et al., 2014). From the above discussion, AA mechanism revolves around the genes affecting cell proliferation and cell cycle events; it can be assumed that these genes contribute to sensitivity of the tumor cells to AA.

On the other hand, genes potentially responsible for responsiveness on curcumin treatment were identified and curcumin downregulated, i.e., *AK2, PDK1, NR2F2, DFFB, MTHFD2*, and *ALDH3B1*. Adenylate kinases (AKs) represent enzymes that catalyze reversible high-energy phosphoryl transfer reactions between adenine nucleotides in the intermembrane space. During periods of metabolic stress, AK2 increases the amount of available adenosine monophosphate and therefore activates downstream ATP-sensing mechanisms—such as AMP-activated protein kinase (AMPK)—to regulate the cellular metabolism. Inhibition of AK2 expression significantly inhibited the proliferation of cancer cells (Dzeja and Terzic, 2009). PDK1 plays a key role in several cancer types. Alterations of PDK1 are critical for oncogenic PI3K signaling. PDK1 has an essential role in regulating cell migration, especially in the context of PTEN deficiency. Downregulation of PDK1 levels inhibits migration and metastasis. PDK1 inhibitors may be useful to prevent cancer progression and abnormal tissue dissemination (Raimondi and Falasca, 2011). The nuclear receptor subfamily 2, group F, member 2 (NR2F2) is a master regulator of angiogenesis and acts as oncogene in prostate and other human cancers. NR2F2 is robustly expressed in the stroma of healthy ovary with little or no expression in epithelia lining the ovarian surface, clefts, or crypts. The pattern of NR2F2 expression was severely disrupted in ovarian cancers, in which decreased levels of stromal expression and ectopic epithelial expression were exhibited. Targeting NR2F2 expression in ovarian cancer cell lines enhanced apoptosis and increased proliferation (Hawkins et al., 2013). *DFFB* contributes to both chromosomal condensation and DNA degradation during apoptosis, decreased *DFFB* expression favors DNA damage, which in turn may contribute to both tumorigenesis and better response to DNA damaging chemotherapy (McDonald et al., 2005). *MTHFD2* mRNA and protein expression is markedly elevated in many cancers and correlated with poor survival in breast cancer. *MTHFD2* is integral to mitochondrial one-carbon metabolism, a metabolic system recently implicated in rapid cancer cell proliferation. Synthesis of one-carbon units carried by the tetrahydrofolate (THF) cofactor is important for proliferating cells, required for nucleotide synthesis and methylation reactions. *MTHFD2* is a bifunctional enzyme, catalyzing the NAD^+^ dependent CH2-THF dehydrogenase and CH^+^-THF cyclohydrolase reactions within the mitochondria. Within the mitochondrial folate pathway, *MTHFD2* is of special interest, because *MTHFD2* was one of the most consistently overexpressed mRNAs genome-wide across 19 different tumor types. The MTHFD2 protein is specifically expressed in transformed cells, but not the stroma surrounding the tumor tissues. MTHFD2 by RNAi impairs proliferation in a variety of cancer cell lines, independent of the tissue of origin, and decreases invasion and migration in breast cancer cell lines. MTHFD2 is broadly required for cancer cell proliferation and viability (Lehtinen et al., 2013; Nilsson et al., 2014). ALDH3B1 is a metabolically active enzyme with distinct specificity for various aldehyde substrates, particularly medium-, and long-chain aliphatic aldehydes. These substrates include many products that are formed during LPO, such as hexanal, 4-hydroxy-2-nonenal (4-HNE), octanal, and trans-2-nonenal. ALDH3B1 plays an important physiological role against cellular oxidative stress by detoxifying aldehydes derived from oxidative processes, such as ethanol metabolism and LPO (Marchitti et al., 2010). Our pharmacogenomics data shows curcumin suppressing cell proliferation by downregulation of anti-apoptotic genes and cell surface adhesion molecules. Curcumin is also seen to regulate cellular metabolism and inhibition of angiogenic cytokines. We can then suggest that the downregulated genes affect the sensitivity of the tumor cells to curcumin.

Previously, we reported the mRNA expression profile induced by curcumin in the NCI cell line panel, which was merged from four different microarray platforms (Novartis, Stanford, Chiron, and Genelogic; Sertel et al., 2012). In the present investigation, we focused only on the Novartis microarray platform for the comparison of curcumin and vitamin C and to reduce the degree of complexity. If we compared the top ranked genes in the previous analysis with those of the present investigation, we found some genes in common (*MTHFD2, AK2, NFATC21P, BTAF1, RBBP4*), although the majority of genes were different in both analyses. This result points to an observation that was frequently made by many investigators: different microarray platforms deliver different results. Nevertheless, several biological functional groups were found to be common in our previous and the present paper, *e.g*. cell cycle, DNA damage response, cell migration, inflammation, signaling pathways, and apoptosis-regulating genes. To our opinion, microarray data are reliable, if they are used for the generation of testable hypotheses. In this respect, both of our microarray analyses were useful. Microarray data represents the starting point for the elucidation of modes of action of cytotoxic compounds rather than completed end results.

Cluster analyses were applied in the present investigation under the assumption that responsiveness of cancer cells might be predicted by using gene expression patterns and that appropriate gene expression profiles might be sufficient to predict whether a cancer cell line is sensitive or resistant to a cytotoxic compound (Sertel et al., 2010). Curcumin revealed two clusters with predominantly sensitive and three with predominantly resistant cell lines. For AA cluster analysis revealed two clusters containing mainly resistant and two clusters containing mainly sensitive cell lines in a comparable fashion to curcumin. The prediction of sensitivity or resistance to cytotoxic drugs by mRNA expression profiles is interesting in the context of individualized or precision medicine, because it may open the possibility to determine prior to treatment, whether or not a tumor will respond to specific drugs. Our data demonstrate that this may not only be feasible to established anticancer drugs, but also to investigative natural products such as curcumin or AA.

The fact that the mRNA expression profiles induced by curcumin and AA related to different gene expression patterns may be related to different modes of actions of both compounds. Medicinal herbs generally contain mixtures of active compounds, which may interact in an additive or synergistic manner. Synergistic interactions may need common mechanisms e.g., a common specific pathway that they inhibit. From an evolutionary point of view, synergistic interactions need co-evolutionary selection pressures to evolve. Hence, it can be speculated that synergisms are less likely to occur than additive effects. Therefore, additive drug interactions can be more frequently found in medicinal herbs. Compounds with different modes of action can efficiently and sufficiently fulfill the requirements for plants to survive under specific evolutionary selection pressure. This may explain that we found additive rather than synergistic interactions in isobologram analyses between curcumin and AA in the panel of cell lines tested. This observation is in accordance with previous data with several cytotoxic compounds from *Artemisia annua* L., which also showed additive rather than synergistic interactions (Efferth et al., 2011).

In summary, we have identified some genes which were downregulated by AA and curcumin. The genes may be responsible for cellular responsiveness of the varied cancer cells to AA and curcumin treatments. The cellular activities tackled by the downregulated genes include inhibition of cell proliferation and cell cycle activities, reduction in cellular metabolism, downregulation of anti-apoptotic gene products and inhibition of angiogenic cytokines. The two natural products seem to induce cytotoxicity by different mechanisms and this may lead to achieve tumor eradication *in vivo*. The varied cellular functionalities represented by the genes downregulated may support additive effects observed in the isobologram analyses.

## Author contributions

Title selection and research design: TE, EO. Laboratory experiments, results generation, data analysis, and interpretation: EO, OK. Manuscript writing and submission: EO. Manuscript writing: HG. Advising in research design, results interpretation, and manuscript writing: TE.

### Conflict of interest statement

The authors declare that the research was conducted in the absence of any commercial or financial relationships that could be construed as a potential conflict of interest.

## Abbreviations

AA: ascorbic acid
IC_50_: 50% inhibitory concentration
NCI: National Cancer Institute.
