# Supplementary material for: Pharmacogenomic Characterization and Isobologram Analysis of the Combination of Ascorbic Acid and Curcumin—Two Main Metabolites of Curcuma longa—in Cancer Cells
Source: Front Pharmacol. 2017 Feb 2;8:38. doi: 10.3389/fphar.2017.00038 (PMC5288649; doi:10.3389/fphar.2017.00038)
Supplement: Supplementary file 1 [file Table1.DOCX]

**Supplementary Table 1**. Phytochemical constituents of three different *Curcuma* species. Data were taken from Dr. Duke’s Phytochemical and Ethnobotanical Databases (<http://www.arsgrin.gov/cgi-bin/duke/farmacy2.pl>).

|  | ***C. longa*** | ***C. zedoaria*** | ***C. xanthorrhiza*** |
| --- | --- | --- | --- |
| **1,4-CINEOLE** | 0 | 1 | 0 |
| **1,8-CINEOLE** | 1 | 1 | 0 |
| **2-BORNANOL** | 1 | 0 | 0 |
| **2-HYDROXY-METHYL-ANTHRAQUINONE** | 1 | 0 | 0 |
| **4-HYDROXY-CINNAMOYL-(FERULOYL)-METHANE** | 1 | 0 | 0 |
| **ALPHA-ATLANTONE** | 1 | 0 | 0 |
| **ALPHA-PHELLANDRENE** | 0 | 0 | 1 |
| **ALPHA-PINENE** | 1 | 1 | 0 |
| **ALPHA-TERPINEOL** | 1 | 0 | 0 |
| **AMYLOSE** | 0 | 1 | 0 |
| **ARABINOSE** | 1 | 0 | 0 |
| **AR-CURCUMENE** | 0 | 0 | 1 |
| **AR-TURMERONE** | 1 | 0 | 0 |
| **ASCORBIC-ACID** | 1 | 0 | 0 |
| **ATLANTONE** | 0 | 0 | 1 |
| **AZULENE** | 1 | 0 | 0 |
| **BETA-CAROTENE** | 1 | 0 | 0 |
| **BETA-CURCUMENE** | 0 | 0 | 1 |
| **BETA-PINENE** | 1 | 0 | 0 |
| **BETA-SESQUIPHELLANDRENE** | 1 | 0 | 0 |
| **BIS-(PARA-HYDROXY-CINNAMOYL)-METHANE** | 1 | 0 | 0 |
| **BISABOLENE** | 1 | 0 | 0 |
| **BIS-DESMETHOXYCURCUMIN** | 1 | 1 | 0 |
| **BORNEOL** | 1 | 0 | 1 |
| **CAFFEIC-ACID** | 1 | 0 | 0 |
| **CALCIUM** | 1 | 0 | 0 |
| **CALCIUM-OXALATE** | 0 | 0 | 1 |
| **CAPRYLIC-ACID** | 1 | 0 | 0 |
| **CARBOHYDRATES** | 1 | 0 | 0 |
| **CARYOPHYLLENE** | 1 | 0 | 0 |
| **CINEOLE** | 1 | 0 | 0 |
| **CINNAMIC-ACID** | 1 | 0 | 0 |
| **CUMINYL-ALCOHOL** | 1 | 0 | 0 |
| **CURCULONE** | 0 | 1 | 0 |
| **CURCUMADIOL** | 0 | 1 | 0 |
| **CURCUMANOLIDE-A** | 0 | 1 | 0 |
| **CURCUMANOLIDE-B** | 0 | 1 | 0 |
| **CURCUMENE** | 1 | 0 | 0 |
| **CURCUMENOL** | 1 | 1 | 0 |
| **CURCUMENONE** | 0 | 1 | 0 |
| **CURCUMIN** | 1 | 1 | 1 |
| **CURCUMOL** | 0 | 1 | 0 |
| **CURDIONE** | 1 | 1 | 0 |
| **CURLONE** | 1 | 0 | 0 |
| **CURZERENONE** | 1 | 1 | 0 |
| **CURZERENONE-C** | 1 | 0 | 0 |
| **CYCLO-ISOPRENEMYRCENE** | 1 | 0 | 0 |
| **D-ALPHA-PHELLANDRENE** | 1 | 0 | 0 |
| **D-ALPHA-PINENE** | 0 | 1 | 0 |
| **D-BORNEOL** | 0 | 1 | 0 |
| **D-CAMPHENE** | 1 | 1 | 0 |
| **D-CAMPHOR** | 1 | 1 | 1 |
| **DEHYDROCURDIONE** | 0 | 1 | 0 |
| **DEHYDROTURMERONE** | 1 | 0 | 0 |
| **DESMETHOXYCURCUMIN** | 1 | 1 | 1 |
| **DICINNAMOYLMETHANE** | 1 | 0 | 0 |
| **DIDESMETHOXYCURCUMIN** | 1 | 0 | 0 |
| **DIFERULOYL-METHANE** | 1 | 1 | 0 |
| **DIHYDROCURCUMIN** | 1 | 0 | 0 |
| **DI-P-COUMAROYL-METHANE** | 1 | 1 | 0 |
| **D-SABINENE** | 1 | 0 | 0 |
| **ENT-CURZERENONE** | 0 | 1 | 0 |
| **EPICURZERENONE** | 0 | 1 | 0 |
| **ETHYL-P-METHOXYCINNAMATE** | 0 | 1 | 0 |
| **EUGENOL** | 1 | 0 | 0 |
| **FERULOYL-P-COUMAROYL-METHANE** | 1 | 1 | 0 |
| **FURANODIENE** | 0 | 1 | 0 |
| **FURANODIENONE** | 0 | 1 | 0 |
| **GAMMA-ATLANTONE** | 1 | 0 | 0 |
| **GERMACRONE-4,5-EPOXIDE** | 0 | 1 | 0 |
| **GUAIACOL** | 1 | 0 | 0 |
| **ISOBORNEOL** | 1 | 0 | 0 |
| **ISOCURCUMENOL** | 0 | 1 | 0 |
| **ISOCURZERENONE** | 0 | 1 | 0 |
| **ISOFURANODIENONE** | 0 | 1 | 0 |
| **ISOFURANOGERMACRENE** | 0 | 1 | 1 |
| **L-ALPHA-CURCUMENE** | 1 | 0 | 0 |
| **L-BETA-CURCUMENE** | 1 | 0 | 0 |
| **L-CYCLOISOPRENEMYRCENE** | 0 | 0 | 1 |
| **LIMONENE** | 1 | 0 | 0 |
| **LINALOL** | 1 | 0 | 0 |
| **MONODESMETHOXYCURCUMIN** | 1 | 0 | 1 |
| **NIACIN** | 1 | 0 | 0 |
| **O-COUMARIC-ACID** | 1 | 0 | 0 |
| **P,P'-DIHYDROXYDICINNAMYOLMETHANE** | 0 | 1 | 0 |
| **P-COUMARIC-ACID** | 1 | 0 | 0 |
| **P-CYMENE** | 1 | 0 | 0 |
| **P-HYDROXYCINNAMOYLFERULOYLMETHANE** | 0 | 1 | 0 |
| **P-METHOXY-CINNAMIC-ACID** | 1 | 0 | 0 |
| **PROCURCUMENOL** | 0 | 1 | 0 |
| **PROTOCATECHUIC-ACID** | 1 | 0 | 0 |
| **P-TOLYMETHYCARBINOL** | 0 | 0 | 1 |
| **P-TOLYMETHYLCARBINOL** | 1 | 0 | 0 |
| **PYROCURCUMENONE** | 0 | 1 | 0 |
| **PYROCURZERENONE** | 0 | 1 | 0 |
| **RIBOFLAVIN** | 1 | 0 | 0 |
| **SESQUITERPENE-ALCOHOLS** | 0 | 1 | 0 |
| **SESQUITERPENES** | 0 | 1 | 0 |
| **SYRINGIC-ACID** | 1 | 0 | 0 |
| **TERPINENE** | 1 | 0 | 0 |
| **TERPINEOL** | 1 | 0 | 0 |
| **THIAMIN** | 1 | 0 | 0 |
| **TURMERONE** | 1 | 0 | 1 |
| **UKONAN-A** | 1 | 0 | 0 |
| **UKONAN-B** | 1 | 0 | 0 |
| **UKONAN-C** | 1 | 0 | 0 |
| **UKONAN-D** | 1 | 0 | 0 |
| **VANILLIC-ACID** | 1 | 0 | 0 |
| **XANTHORRHIZOL** | 0 | 0 | 1 |
| **ZEDERONE** | 0 | 1 | 0 |
| **ZEDOARONE** | 0 | 1 | 0 |
| **ZINGIBERENE** | 1 | 0 | 1 |
| **ZINGIBEROL** | 0 | 0 | 1 |
